# Supplementary figures and images for: Structural Variants and Implicated Processes Associated with Familial Tourette Syndrome
Source: Int J Mol Sci. 2024 May 25;25(11):5758. doi: 10.3390/ijms25115758 (PMC11171586; doi:10.3390/ijms25115758)

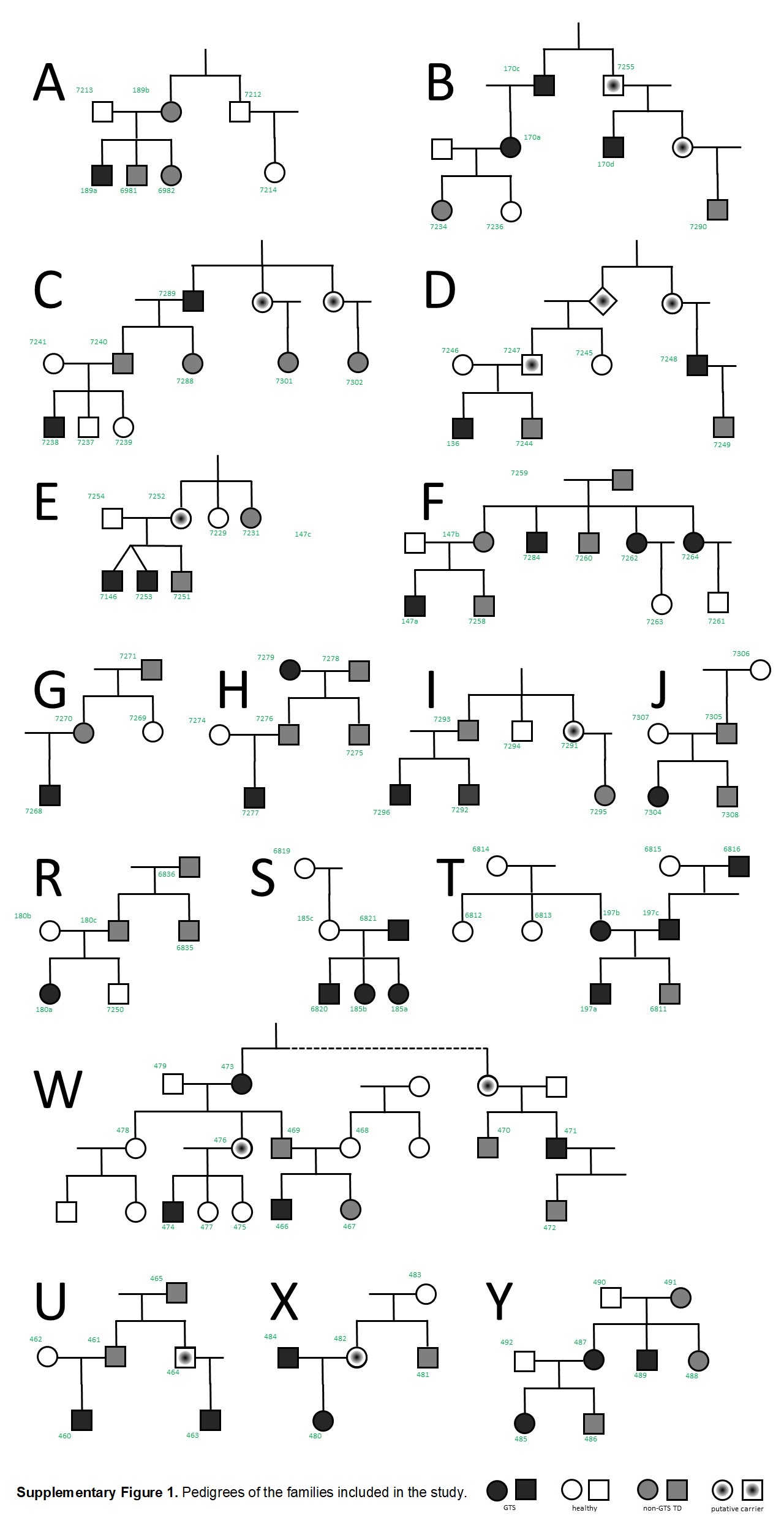

Supplement: Supplementary file 1 [file ijms-25-05758-s001.zip › FigureS1.jpg]
